# Genomic Characterization and Biotechnological Potential of a Skatole-Degrading *Bacillus pumilus* Strain hhy901 Isolated from Bovine Manure

**DOI:** 10.3390/cimb48090964

**Published:** 2026-09-20

**Authors:** Zhonghao Wang, Wenjie Zhang, Weibing Zhang, Wei Zhang, Yulong Zhao, Feier Ren, Kaili Zhang, Jie Cheng, Jiajin Sun, Hongyan Hou

**Affiliations:** 1 Key Laboratory of Livestock and Poultry Healthy Breeding Technology in Northwest China, Ministry of Agriculture and Rural Affairs, College of Animal Science and Technology, Xinjiang Agricultural Vocational and Technical University, Changji 831100, China; 2 College of Animal Science and Technology, Northeast Agricultural University, Harbin 150030, China

**Keywords:** *Bacillus pumilus*, bioremediation, environmental biotechnology, functional annotation, livestock manure deodorization, skatole, whole-genome sequencing

## Abstract

Skatole (3-methylindole) is a primary malodorous compound emitted from livestock manure, posing significant challenges for odor control in intensive animal production. In this study, a skatole-degrading bacterial strain, designated hhy901, was isolated from fresh cattle manure and identified as *Bacillus pumilus* through polyphasic taxonomy, including 16S rRNA and gyrB gene phylogeny, morphological characterization, biochemical profiling, and whole-cell fatty acid analysis. Under an initial skatole concentration of 100 mg/L in *Bacillus* liquid medium, strain hhy901 removed 87.2% of skatole within 96 h, as determined by high-performance liquid chromatography (HPLC). Whole-genome sequencing revealed a genome of approximately 3.60 Mb with a GC content of 41.91%, encoding 3564 predicted protein-coding genes. Functional genomic annotation identified 39 genes associated with xenobiotic biodegradation and metabolism, including putative catechol dioxygenase, aldehyde dehydrogenase, and cytochrome P450 genes, which together suggest a potential skatole catabolic pathway. Furthermore, the genome harbored a broad repertoire of genes associated with resistance to heavy metals, disinfectants, and multiple antibiotic classes, indicating a potential for environmental stress adaptation. Virulence factor profiling predominantly identified genes related to nutrient acquisition and microbial competition, with only a single invasion-associated factor detected, suggesting a relatively low invasive pathogenic potential. However, the presence of multiple antimicrobial resistance determinants warrants cautious interpretation, and phenotypic validation of both degradation function and biosafety is required before environmental application. These results establish *B. pumilus* hhy901 as a promising microbial candidate for further development of bio-deodorization strategies in livestock production, pending functional and safety validation.

## 1. Introduction

Skatole (3-methylindole) is the most representative indolic odorant in livestock manure, generated through the anaerobic metabolism of tryptophan by gut microbiota. Owing to its extremely low olfactory threshold and persistent malodor, skatole has become a core target pollutant for odor control in animal farming [1]. Conventional deodorization methods, such as physical adsorption and chemical oxidation, often suffer from high operational costs and the risk of secondary pollution. By contrast, microbial degradation has emerged as the dominant technological pathway for livestock manure odor mitigation due to its environmental friendliness, low cost, and capacity for in situ remediation. The isolation of functional microbial strains with efficient skatole-degrading capabilities is therefore a critical prerequisite for the practical implementation of this biotechnology [2,3].

A growing body of literature has documented the diversity of skatole-degrading microorganisms isolated from various environmental matrices, including livestock manure, rumen fluid, activated sludge, and contaminated soils. A recent comprehensive review by Xu et al. [4] summarized the current advances in microbial skatole degradation, covering bacterial genera such as *Pseudomonas*, *Acinetobacter*, *Sphingobacterium*, *Lactobacillus*, and *Bacillus*, as well as the proposed degradation pathways involving hydroxylation, ring cleavage, and subsequent mineralization. Among these, strains of the genus *Bacillus* have attracted particular attention due to their ability to form endospores, which confers exceptional tolerance to heat, desiccation, and environmental stress—properties highly advantageous for the formulation and storage of bio-inoculants for field application. However, compared with other genera, the number of *Bacillus* strains explicitly characterized for skatole degradation remains limited. For instance, Wang et al. [5] isolated skatole-degrading bacteria from bovine rumen and reported a *Bacillus* strain that achieved 23.03% degradation of 100 mg/L skatole within 48 h. Xu et al. [6] screened deodorizing strains from chicken manure and identified a *Bacillus* isolate capable of removing 44.5% of 10 mg/L skatole within 24 h. In addition, dietary supplementation with *Bacillus* subtilis has been shown to modulate intestinal microbiota and reduce odorant emissions in weaned piglets [7], further supporting the potential of *Bacillus*-based approaches for manure deodorization. Nevertheless, the skatole degradation efficiencies reported for *Bacillus* strains to date remain relatively modest, and the genomic basis underlying their degradation capacity and environmental adaptability remains poorly characterized.

Members of the genus *Bacillus* are characterized by strong environmental adaptability, the ability to form endospores that confer tolerance to high temperatures, heavy metals, disinfectants, and other stresses, and the genetic potential to secrete various oxidases and hydrolases, making them widely applicable in the biodegradation of organic pollutants [8]. As a typical species of this genus, *Bacillus pumilus* has been extensively studied for plant growth promotion and disease antagonism, but its potential for environmental bioremediation—particularly the degradation of indolic malodorants—remains underexplored. Systematic investigations into its skatole-degrading function and underlying molecular mechanisms are still lacking, and comprehensive genomic evidence for its biotechnological application is limited [9]. Moreover, residual heavy metals, various veterinary antibiotics, and disinfectants in livestock manure can severely inhibit the survival and functionality of degrading bacteria; therefore, the isolation of indigenous strains that combine high degradation efficiency with broad stress resistance is crucial for practical applications in manure environments [10].

Polyphasic taxonomy represents the standard system for accurate prokaryotic species delineation, integrating colony and cell morphology, chemotaxonomy (whole-cell fatty acid profiling), physiological and biochemical characteristics, and single- or multi-locus housekeeping gene phylogeny, thereby effectively avoiding the identification bias inherent in any single method [11]. While 16S rRNA gene sequencing remains the cornerstone of bacterial identification, closely related *Bacillus* species often exhibit >99% 16S rRNA sequence similarity, necessitating the use of additional housekeeping genes (e.g., *gyrB*, *rpoB*, *recA*) and, ideally, whole-genome-based methods such as average nucleotide identity (ANI) for definitive species assignment [12]. High-throughput whole-genome sequencing and bioinformatic annotation can further elucidate the functional repertoire of a strain at the genetic level, including carbon metabolism, aromatic pollutant degradation, environmental stress resistance, and biosafety, thereby revealing the molecular basis of skatole degradation and the mechanisms of environmental adaptation [13]. These genomic approaches are essential for evaluating the biotechnological potential of newly isolated strains.

In this study, we aimed to (i) isolate and identify a skatole-degrading *B. pumilus* strain from cattle manure using polyphasic taxonomy, supplemented by genome-based taxonomic comparisons; (ii) evaluate its skatole degradation efficiency under controlled laboratory conditions; (iii) characterize its genome through high-throughput sequencing and comprehensive bioinformatic annotation; and (iv) annotate genes putatively involved in xenobiotic degradation, stress resistance, and biosafety to assess its biotechnological potential for manure deodorization. The results provide a microbial resource and genomic framework for the development of bio-based deodorization technologies in livestock production, while also identifying key knowledge gaps requiring future functional validation.

## 2. Materials and Methods

### 2.1. Chemicals and Reagents

*Bacillus* Medium (HB8786, Hopebio, Qingdao, China), sterile Petri dishes, absolute ethanol, a LiChrospher^®^ RP-18 HPLC column (5 μm particle size, 25 cm × 4.6 mm i.d., Sigma-Aldrich, St. Louis, MO, USA), acetonitrile (HPLC grade), methanol (HPLC grade), 2-methylindole (2-MI, Macklin, Shanghai, China), Tris buffer, 0.22 μm organic membrane filters, syringes, 3-methylindole (3-MI, skatole, Macklin, Shanghai, China), 2 mL autosampler vials, and sterile physiological saline were used.

### 2.2. Isolation and Screening of Skatole-Degrading Bacteria

Fresh cattle manure (100 g) was collected from Changji (China) and suspended in 900 mL sterile physiological saline. The suspension was shaken at 37 °C and 100 rpm for 1 h to release endogenous microorganisms. The suspension was serially diluted (10^−1^ to 10^−6^), and 100 μL aliquots of each dilution were spread onto *Bacillus* selective agar plates containing 100 mg/L skatole. After incubation at 37 °C for 24 h, ten morphologically distinct single colonies were picked and individually enriched in 5 mL *Bacillus* liquid medium at 37 °C, 180 rpm for 24 h. The isolates were further purified by streak plating on *Bacillus* agar.

For degradation capacity screening, each purified isolate was inoculated into 100 mL *Bacillus* liquid medium supplemented with 100 mg/L skatole in 250 mL Erlenmeyer flasks, at an initial optical density at 600 nm (OD_600_) of 0.1. The cultures were incubated at 37 °C with shaking at 180 rpm for 96 h. Uninoculated *Bacillus* liquid medium containing 100 mg/L skatole was included as an abiotic control to account for volatilization and adsorption losses. All experiments were performed in 3 independent biological replicates. After 96 h, residual skatole concentrations were determined by HPLC as described in Section 2.3. The strain exhibiting the highest skatole removal was designated hhy901 and selected for subsequent polyphasic taxonomic identification at the Agricultural Culture Collection of China (ACCC) and whole-genome sequencing performed by Shanghai Majorbio Bio-pharm Technology Co., Ltd.

### 2.3. Skatole Quantification

For detailed degradation kinetics, strain hhy901 was cultured in *Bacillus* liquid medium containing 100 mg/L skatole under the same conditions described in Section 2.2. Samples (2 mL) were collected at 0, 12, 24, 48, 72, and 96 h. Each sample was centrifuged at 12,000× *g* for 10 min at 4 °C, and the supernatant was filtered through a 0.22 μm organic membrane filter into 2 mL autosampler vials for HPLC analysis.

Skatole concentration was determined by high-performance liquid chromatography (HPLC) using an Agilent 1260 Infinity II system equipped with a LiChrospher^®^ RP-18 column (5 μm, 25 cm × 4.6 mm i.d., Sigma-Aldrich, St. Louis, MO, USA) and a fluorescence detector (FLD). The fluorescence detection conditions were set at an excitation wavelength of 270 nm and an emission wavelength of 352 nm. The mobile phase consisted of water, acetonitrile, and methanol at a ratio of 50:40:10 (*v*/*v*/*v*). The column temperature was maintained at 35 °C, the flow rate was set at 1.0 mL/min, and the injection volume was 10 μL. The total run time for each sample was 15 min. For accurate quantification, 2-methylindole (2-MI) was used as an internal standard to minimize operational errors introduced during sample processing and improve the reliability of the results. The internal standard solution was prepared as 0.05 M Tris buffer containing 10 μg/mL 2-MI. Skatole was quantified using an internal standard calibration curve prepared from authentic skatole standards at concentrations of 1, 5, 10, 20, 50, 100, and 200 μg/mL [14].

Sample pretreatment prior to HPLC analysis was performed as follows: (1) 100 μL of the culture supernatant was mixed with 400 μL of HPLC-grade acetonitrile; (2) 500 μL of 0.05 M Tris buffer containing 10 μg/mL 2-MI (internal standard) was added; (3) the mixture was thoroughly vortexed and filtered through a 0.22 μm organic membrane filter to obtain the sample for injection. The degradation percentage was calculated as:

Degradation (%) = [(C_0_ − C_t_)/C_0_] × 100%

where C_0_ is the initial skatole concentration and C_t_ is the residual skatole concentration at time t. Values were corrected for abiotic losses measured in the uninoculated control. The degradation rate (mg/L/h) was calculated as the total mass of skatole degraded divided by the incubation time. All data are presented as mean ± standard deviation (SD) of 3 biological replicates. Statistical significance was assessed by one-way ANOVA followed by Tukey’s post hoc test using SPSS 26.0, with *p* < 0.05 considered statistically significant (Supplementary Table S1).

### 2.4. 16S rRNA and gyrB Gene Amplification and Sequencing

The 16S rRNA and gyrB gene amplification and sequencing were performed by the Agricultural Culture Collection of China (ACCC). The 16S rRNA gene was amplified using the universal bacterial primers 27F (5′-GTTTGATCMTGGCTCAG-3′) and 1492R (5′-TACGGYTACCTTGTTACGACTT-3′). The *gyrB* gene fragment was amplified with the degenerate primers UP-1 (5′-GAAGTCATCATGACCGTTCTGCAYGCNGGNGGNAARTTYGA-3′) and UP-2r (5′-AGCAGGATACGGATGTGCGAGCCRTCNACRTCNGCRTCNGTCAT-3′). PCR products were purified and sequenced bidirectionally. PCR products were purified and sequenced bidirectionally by the ACCC.

### 2.5. Morphological and Staining Characteristics

Morphological and staining characterization was performed by the ACCC. Strain hhy901 was streaked onto Trypticase Soy Agar (TSA) and incubated at 30 °C for 24 h. Colony morphology (shape, elevation, margin, surface wrinkling, and color) was observed and photographed. Gram staining and endospore staining were performed, and cell morphology, Gram reaction, and spore formation were examined under a light microscope.

### 2.6. Physiological and Biochemical Characterization

Physiological and biochemical characterization, including API 50CH carbon source utilization and whole-cell fatty acid analysis, was performed by the ACCC. Carbon source utilization was tested using the API 50CH system (bioMérieux, Marcy-l’Étoile, France) following the manufacturer’s instructions. Results were recorded as positive (+), weakly positive (w), or negative (−). For whole-cell fatty acid analysis, strain hhy901 was grown in Tryptic Soy Broth to the stationary phase, and fatty acids were extracted, saponified, methylated, and identified by gas chromatography using the MIDI Sherlock Microbial Identification System. Relative percentages of fatty acids were compared with the typical profile of the genus *Bacillus* for chemotaxonomic assignment.

### 2.7. Whole-Genome Sequencing and Bioinformatics

Whole-genome sequencing was performed by Shanghai Majorbio Bio-pharm Technology Co., Ltd. (Shanghai, China) using a combined PacBio and Illumina sequencing strategy. For PacBio single-molecule real-time (SMRT) sequencing, genomic DNA was sheared to 8–10 kb fragments using G-tubes. Sequencing was performed on a PacBio Sequel II platform. For Illumina sequencing sequenced on an Illumina NovaSeq 6000 platform with 2 × 150 bp paired-end reads (data were deposited in NCBI under accession number SAMC8439660).

Raw PacBio reads were filtered and assembled de novo using Flye v2.9.2 (https://github.com/mikolmogorov/Flye (accessed on 31 August 2026)). The resulting assembly was polished using Pilon v with Illumina short reads to correct base-level errors. Illumina raw reads were quality-filtered using fastp v0.20.0 (https://github.com/OpenGene/fastp (accessed on 31 August 2026)) with the following criteria: removal of adapter sequences, trimming of bases with quality score < 20, and discarding reads with length < 50 bp.

Gene prediction was carried out with Prodigal v for the chromosome, identifying a total of 3564 protein-coding sequences. Transfer RNAs were predicted using tRNAscan-SE, and ribosomal RNAs were predicted using Barrnap. Functional annotation was conducted by BLASTP searches (e-value threshold: 1 × 10^−5^) against the NCBI Non-Redundant (NR) database, Swiss-Prot, Pfam, Clusters of Orthologous Groups (COG), Kyoto Encyclopedia of Genes and Genomes (KEGG), and Gene Ontology (GO) databases.

Antibiotic resistance genes were identified using the Comprehensive Antibiotic Resistance Database (CARD, Version 1.1.3) and ResFinder (https://cge.cbs.dtu.dk/services/ResFinder/ (accessed on 31 August 2026)). Heavy metal and disinfectant resistance genes were annotated using the BacMet database. Virulence factors were identified by BLAST (version 2.17.0) searches against the Virulence Factor Database (VFDB). Mobile genetic elements, including genomic islands (GIs), prophages, insertion sequences (IS), integrons, and CRISPR-Cas systems, were predicted using the Majorbio cloud platform. For genome-based taxonomic confirmation, average nucleotide identity (ANI) was calculated between strain hhy901 and closely related *Bacillus* species using the Majorbio cloud platform based on Mash distance and whole-genome comparison.

### 2.8. Data Analysis

Homology searches for 16S rRNA and gyrB gene sequences were performed against the EzBioCloud (https://www.ezbiocloud.net/; accessed on 15 July 2026) and NCBI (https://www.ncbi.nlm.nih.gov/; accessed on 15 July 2026) databases [15]. Phylogenetic trees were reconstructed using the neighbor-joining method in MEGA 11 (jointly developed by Temple University, USA, and Tokyo Metropolitan University, Japan) with 1000 bootstrap replicates. For genome-based taxonomic analysis, the Type (Strain) Genome Server (TYGS, https://tygs.dsmz.de/ (accessed on 31 August 2026)) was employed to calculate pairwise digital DNA–DNA hybridization (dDDH) values using the GGDC 3.0 formula and to infer a whole-genome blast distance phylogeny (GBDP) tree with 100 pseudo-bootstrap replicates [16,17]. The average nucleotide identity (ANI) was calculated using the OrthoANIu algorithm. The taxonomic position of strain hhy901 was determined according to standard polyphasic criteria based on morphological, biochemical, chemotaxonomic, and molecular phylogenetic data, supplemented by genome-based ANI and dDDH comparisons. All figures were generated using GraphPad Prism 9 (GraphPad Software, San Diego, CA, USA) and finalized in Adobe Illustrator (Adobe Systems, San Jose, CA, USA, version 30.7).

## 3. Results

### 3.1. Polyphasic Identification of Strain hhy901

A skatole-degrading bacterial strain, designated hhy901, was isolated from fresh cattle manure based on its ability to remove skatole from liquid culture. Polyphasic taxonomic methods were employed to determine its taxonomic position.

The 16S rRNA gene sequence of strain hhy901 was obtained with a length of 1451 bp. BLAST-based comparison against the EzBioCloud database (http://www.ezbiocloud.net/eztaxon/; accessed on 15 July 2026) revealed that the strain shared the highest sequence similarity (99.93%) with the type strain *Bacillus pumilus* ATCC 7061T. Similarity values to other closely related type strains are listed in Appendix A. The phylogenetic tree based on 16S rRNA gene sequences, constructed with strain hhy901 and related taxa, is shown in Figure S1.

The gyrB gene fragment of strain hhy901 was amplified, yielding a product of 1136 bp. An NCBI BLAST search indicated that this sequence exhibited the highest similarity (97.50%) with that of *Bacillus pumilus* ATCC 7061T. The phylogenetic tree inferred from gyrB gene sequences (Figure S2) placed strain hhy901 in a robust cluster with *B. pumilus* ATCC 7061T, supported by a 100% bootstrap value.

**Figure 1:**
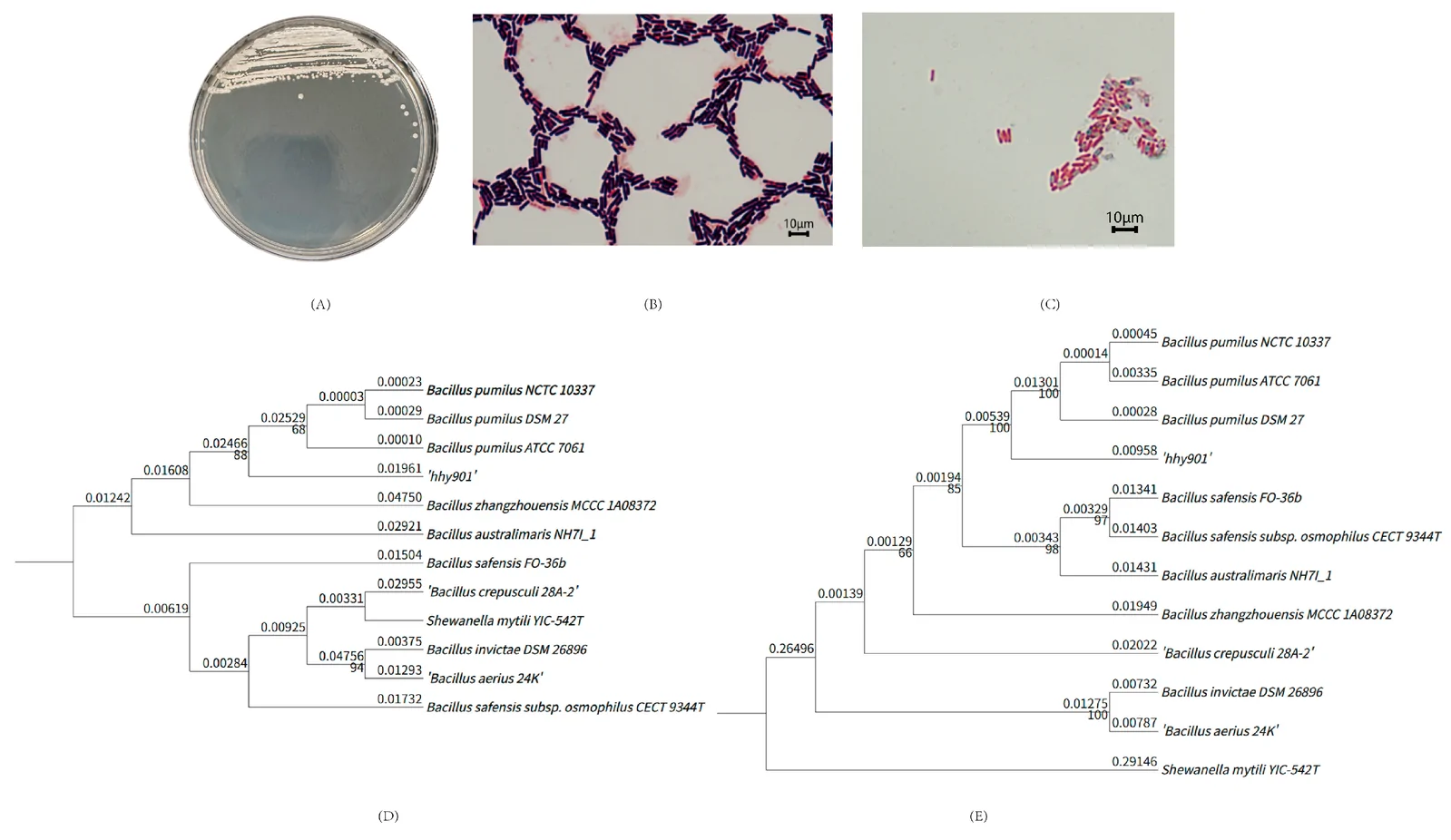
Polyphasic characterization of strain hhy901. (**A**) Colony morphology of strain hhy901 on TSA after 24 h at 30 °C. (**B**) Gram staining showing Gram-positive, rod-shaped cells. (**C**) Endospore staining. (**D**) Whole-genome phylogenomic tree inferred by the Genome Blast Distance Phylogeny (GBDP) method using the Type (Strain) Genome Server (TYGS, (https://tygs.dsmz.de/ (accessed on 31 August 2026))), showing the phylogenetic position of strain hhy901 among species of the *Bacillus pumilus* group and related taxa. Numbers above branches indicate GBDP distance values; numbers at nodes represent pseudo-bootstrap support percentages from 100 replications. (**E**) Whole-proteome-based tree inferred by the Genome Blast Distance Phylogeny (GBDP) method using the Type (Strain) Genome Server (TYGS, (https://tygs.dsmz.de/ (accessed on 31 August 2026))), showing the phylogenetic position of strain hhy901 among species of the *Bacillus pumilus* group and related taxa. Numbers above branches indicate GBDP distance values; numbers at nodes represent pseudo-bootstrap support percentages from 100 replications.

Morphologically, after 24 h of cultivation on TSA at 30 °C, strain hhy901 formed round, convex colonies with regular, wrinkled margins (Figure 1A). The cells were Gram-positive and rod-shaped (Figure 1B). Endospore staining confirmed the formation of terminal or subterminal endospores (Figure 1C). These morphological and staining characteristics are consistent with those typical of the genus *Bacillus*.

To further confirm the species assignment, whole-genome-based taxonomic comparisons were performed. The average nucleotide identity (ANI) between strain hhy901 and *B. pumilus* ATCC 7061 was 95.73%, as determined by OrthoANIu analysis. Digital DNA–DNA hybridization (dDDH) analysis using the Type (Strain) Genome Server (TYGS) yielded a d4 value (GGDC formula 2) of 64.3% with *B. pumilus* ATCC 7061T, with high genome coverage (d0 = 90.7%, d6 = 88.7%). The GBDP tree (whole-genome sequence-based) (Figure 1D) and GBDP tree (whole-proteome-based) (Figure 1E) placed strain hhy901 within the *B. pumilus* clade with strong bootstrap support. Although the d4 value was below the 70% species threshold, the ANI value exceeded the 95% cutoff, and the strain clustered consistently with *B. pumilus* in phylogenomic analysis. Combined with the polyphasic evidence (16S rRNA, gyrB, morphology, biochemistry, and fatty acid profiling), these genome-based data support the assignment of strain hhy901 to the species *B. pumilus*, while indicating a relatively divergent genomic position within the species.

### 3.2. Physiological and Biochemical Characteristics of Strain hhy901

The whole-cell fatty acid profile of strain hhy901, determined using the MIDI Sherlock Microbial Identification System, revealed that the predominant fatty acids were C15:0 anteiso (39.72%), C15:0 iso (29.69%), C17:0 anteiso (9.23%), and C16:0 iso (8.36%). The complete fatty acid composition is presented in Table 1. This profile is consistent with the typical fatty acid pattern of the genus *Bacillus*.

In the API 50CH test, strain hhy901 utilized D-ribose, D-glucose, D-fructose, D-mannose, D-mannitol, arbutin, esculin, salicin, D-sucrose, and D-tagatose (positive reactions); L-arabinose, D-xylose, amygdalin, D-cellobiose, D-trehalose, and gentiobiose were weakly positive. All other tested substrates were negative. The detailed results are provided in Table 2. These metabolic characteristics conform to the biochemical profile of the genus *Bacillus*.

### 3.3. Genome Characteristics of Bacillus pumilus hhy901

As shown in Table 3, whole-genome sequencing revealed that the genome of *Bacillus pumilus* hhy901 consists of a single circular chromosome of 3599,587 bp with a G+C content of 41.91%; no plasmids were detected. Gene prediction identified a total of 3564 protein-coding sequences in the genome.

### 3.4. Functional Annotation of Bacillus pumilus hhy901

**Figure 2:**
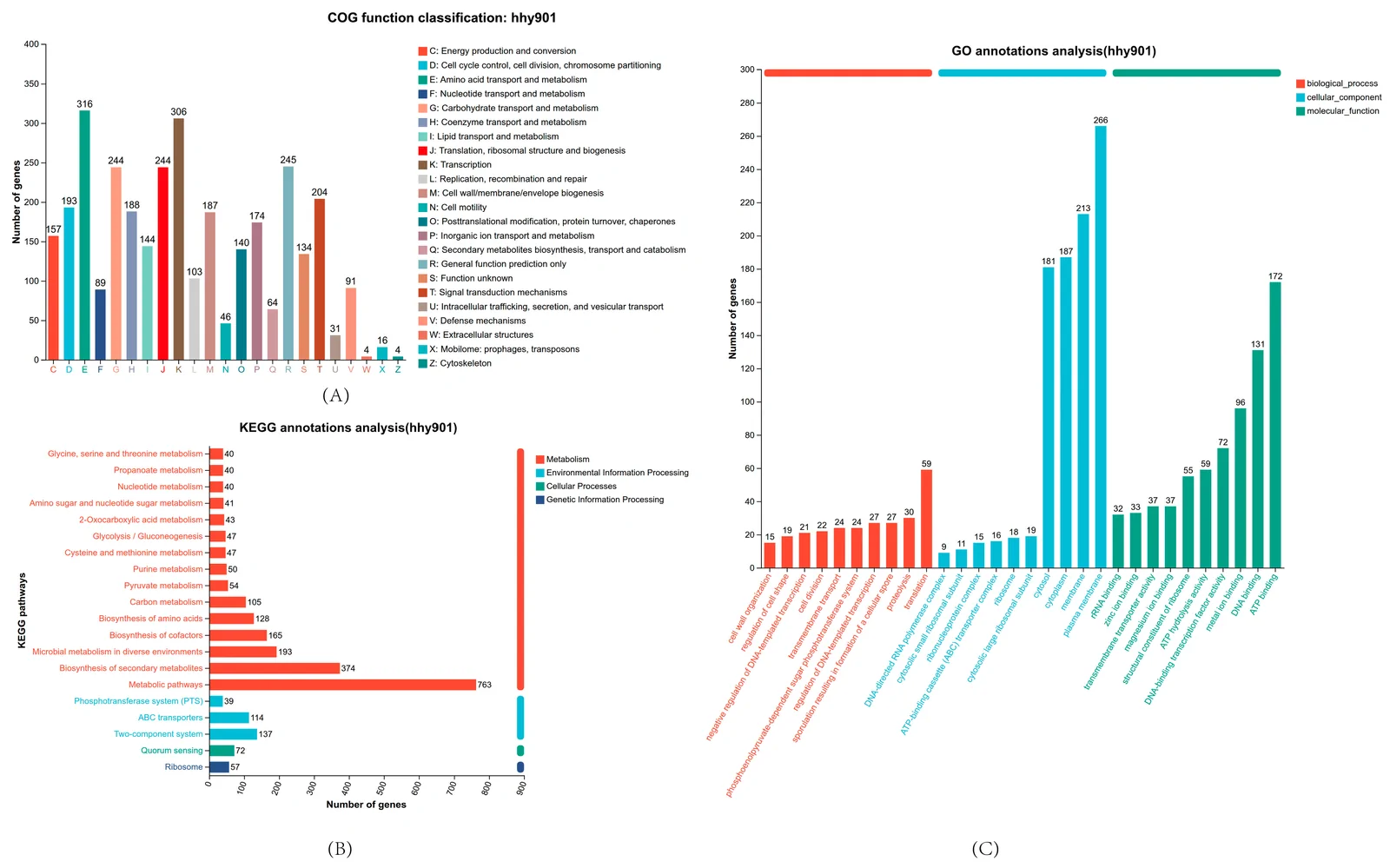
Functional annotation of the *Bacillus pumilus* hhy901 genome. (**A**) COG functional classification showing the distribution of predicted proteins across different functional categories. (**B**) KEGG pathway classification assigning annotated genes to metabolism, cellular processes, genetic information processing, environmental information processing, organismal systems, and human diseases. (**C**) Top 10 enriched Gene Ontology (GO) terms in the categories of biological process, molecular function, and cellular component.

COG functional classification assigned the predicted proteins to 21 functional categories (Figure 2A). The most abundant categories were amino acid transport and metabolism (316 proteins), transcription (306), carbohydrate transport and metabolism (244), translation, ribosomal structure, and biogenesis (244), and general function prediction only (245). The high representation of amino acid and carbohydrate metabolism genes is consistent with the saprophytic lifestyle of *B. pumilus* in nutrient-rich manure environments. Genes associated with defense mechanisms (91), signal transduction (204), and inorganic ion transport (174) were also well represented, reflecting the strain’s capacity for environmental sensing and stress response.

KEGG pathway analysis mapped the annotated genes to major functional categories, including metabolism, cellular processes, genetic information processing, environmental information processing, and organismal systems (Figure 2B). Within the metabolism category, genes involved in carbohydrate metabolism, amino acid metabolism, and energy metabolism were the most numerous. Notably, KEGG annotation also identified genes associated with xenobiotic biodegradation and metabolism, which are analyzed in detail in Section 3.5.

Gene Ontology (GO) analysis classified the predicted genes into three ontologies: biological process (1336 genes), molecular function (2125 genes), and cellular component (1081 genes). The top 10 enriched GO terms are summarized in Figure 2C. In the molecular function category, catalytic activity and binding were the most highly represented terms, consistent with the strain’s broad metabolic capacity.

### 3.5. Genes Associated with Xenobiotic Degradation, Stress Resistance, and Biosafety

#### 3.5.1. Carbohydrate-Active Enzymes

**Figure 3:**
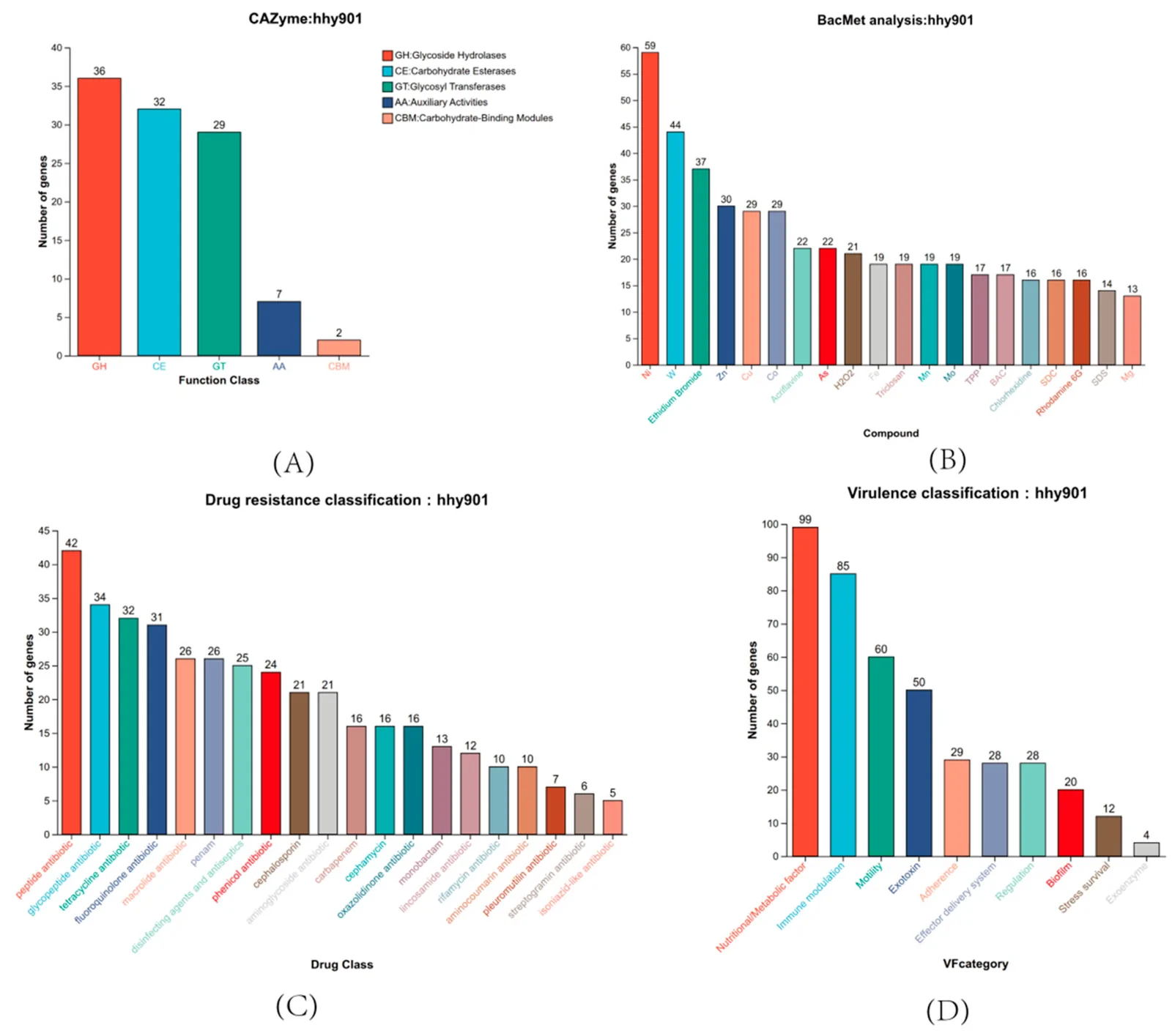
Functional genomic analysis of *Bacillus pumilus* hhy901. (**A**) CAZy carbohydrate-active enzyme classification showing the numbers of genes assigned to glycoside hydrolases (GHs), carbohydrate esterases (CEs), glycosyltransferases (GTs), auxiliary activities (AAs), polysaccharide lyases (PLs), and carbohydrate-binding modules (CBMs). (**B**) BacMet-based annotation of resistance genes to heavy metals, disinfectants, and organic solvents, highlighting the copy numbers of the major stress resistance determinants. (**C**) Whole-genome antibiotic resistance gene profiling across multiple drug classes. (**D**) Virulence factor analysis categorized by functional group (e.g., nutrition/metabolism, immune modulation, motility, exotoxins, adhesion, biofilm formation, stress tolerance, invasion).

Based on the CAZy database, a total of 108 carbohydrate-active enzyme-encoding genes belonging to six major classes were identified in the genome of *B. pumilus* hhy901 (Figure 3A). These comprised 36 glycoside hydrolases (GHs), 32 carbohydrate esterases (CEs), 29 glycosyltransferases (GTs), 7 auxiliary activities (AAs), 2 polysaccharide lyases (PLs), and 2 carbohydrate-binding modules (CBMs). The abundance of GHs and CEs suggests that strain hhy901 possesses a substantial capacity for polysaccharide degradation, which may support its proliferation in manure rich in plant-derived carbohydrates.

#### 3.5.2. Putative Xenobiotic Biodegradation Genes

KEGG functional annotation identified a total of 39 genes associated with xenobiotic biodegradation and metabolism in the genome of *B. pumilus* hhy901 (Supplementary Table S2). These genes are distributed across multiple pathways for aromatic compound degradation, including ring cleavage, aldehyde oxidation, cytochrome P450-mediated oxidation, nitroreduction, and β-oxidation.

Notably, a gene encoding catechol 2,3-dioxygenase (C23O, catE) was detected. C23O catalyzes the meta-cleavage of catechol at the 2,3-position, representing a key enzyme in the aerobic degradation of aromatic compounds, including indolic pollutants [18]. In addition, genes encoding aromatic intermediate-metabolizing enzymes such as 2-hydroxymuconic semialdehyde hydrolase (dmpD), 4-carboxymuconolactone decarboxylase (pcaC), and 4-oxalocrotonate tautomerase (praC), along with key genes of the phenylacetic acid degradation pathway (paaH, atoB, atoD, atoA), were also annotated. Together, these genes constitute a putative downstream metabolic network for aromatic ring processing, which may provide the genetic basis for ring cleavage and eventual mineralization of indolic compounds such as skatole.

With respect to oxidoreductase systems, the genome harbors three copies of the aldehyde dehydrogenase gene (ALDH), which may participate in the degradation of toxic aldehyde intermediates generated during aromatic compound breakdown [18]. A monoamine oxidase gene (aofH) was identified, which is potentially involved in the oxidative deamination of amines and may relate to the initial transformation of nitrogen-containing heterocyclic pollutants. The cytochrome P450 gene (cypD_E) may mediate the hydroxylation of xenobiotics, thereby enhancing pollutant solubility and degradability. Genes of the nitroreductase family (nfsA, nfnB, nbaA) and the N-ethylmaleimide reductase gene (nemA) may collectively contribute to the reductive detoxification of nitroaromatic and quinone-type xenobiotics [19].

Furthermore, genes such as the amidase gene amiE may further expand the strain’s xenobiotic-degradation spectrum [20]. The β-oxidation-related enzymes FadA, FadB, and FadN are potentially involved in the carbon-chain metabolism of degradation products, which may ultimately feed into central carbon metabolic pathways. The systematic presence of these xenobiotic-degradation genes suggests that strain hhy901 possesses a well-equipped genetic system for aromatic pollutant degradation, which is consistent with its observed skatole-degradation phenotype. However, it should be emphasized that these annotations represent genomic potential, and the actual involvement of these genes in skatole degradation requires experimental validation through transcriptomic, proteomic, or enzyme activity analyses.

#### 3.5.3. Putative Stress Resistance Determinants

The genome of *B. pumilus* hhy901 was screened against the BacMet database to identify genes potentially associated with resistance to heavy metals, dyes, disinfectants, oxidants, organic solvents, acids, and alkalis (Figure 3B). A substantial number of stress resistance genes were annotated, with marked differences in copy number among various stressor categories. It should be noted that BacMet annotations include both experimentally confirmed and predicted resistance genes, and some annotations may correspond to general transport proteins with broad substrate specificity rather than dedicated resistance determinants.

Genes associated with heavy metal resistance were the most abundant. Nickel (Ni) resistance-associated genes were the most numerous (59 copies), followed by tungsten (W) (44 copies), zinc (Zn) (30), cobalt (Co) (29), and copper (Cu) (29). Nineteen resistance-associated genes each were found for molybdenum (Mo), manganese (Mn), iron (Fe), and triclosan. Arsenic (As) resistance was represented by 22 genes, whereas copy numbers for mercury (Hg), tellurium (Te), chromium (Cr), selenium (Se), cadmium (Cd), lead (Pb), and other metals were relatively low. The high number of nickel and tungsten resistance genes may partly reflect the presence of multiple paralogous metal transporter systems (e.g., NikABCDE-type permeases) rather than dedicated resistance mechanisms, and their functional significance requires phenotypic validation.

Regarding resistance to dyes and antimicrobial compounds, 37 genes related to ethidium bromide were identified, and corresponding resistance elements were also detected for acriflavine, rhodamine 6G, crystal violet, and acridine orange. Additionally, 21 genes potentially associated with hydrogen peroxide (H_2_O_2_) resistance were annotated, which may enable the strain to scavenge oxidative free radicals and mitigate oxidative damage.

The genome also harbored genes potentially associated with resistance to disinfectants and surfactants commonly used in livestock operations, including benzalkonium chloride (BAC), chlorhexidine, sodium dodecyl sulfate (SDS), cetrimonium bromide (CTM), quaternary ammonium compounds (QACs), Triton X-100, CTAB, and sodium deoxycholate (SDC). This broad complement of resistance-associated genes suggests that the strain may possess a genetic basis for tolerating routine disinfection procedures, which could be advantageous for field application in manure deodorization. However, phenotypic tolerance assays are required to confirm these predictions.

Furthermore, resistance-associated genes against various organic solvents (e.g., n-hexane, toluene, ethylbenzene, xylene, cyclohexane), acids (HCl), alkalis (NaOH), phenols, fatty acids, and ionophores (CCCP, TPP) were identified. In contrast, only one to two resistance-associated genes were found for pentane, sodium azide, spermine, linoleic acid, and dimethyl phthalate, indicating a relatively weaker predicted tolerance toward these compounds.

#### 3.5.4. Antibiotic Resistance Gene Profiling

Whole-genome annotation of antimicrobial resistance genes (Figure 3C) revealed a diverse array of determinants potentially associated with resistance to various antibiotic classes. The most abundant categories were peptide antibiotic resistance genes (42 copies), followed by glycopeptide (34), tetracycline (32), and fluoroquinolone (31) resistance-associated genes. Penam and macrolide resistance-associated genes each accounted for 26 copies. Additionally, 25 genes related to disinfectant and antiseptic resistance were identified.

Resistance-associated genes for chloramphenicol, aminoglycoside, and cephalosporin were present at 24, 21, and 21 copies, respectively. Oxazolidinone, cephamycin, and carbapenem resistance-associated genes each totaled 16 copies. It is important to note that many of these annotations may correspond to conserved housekeeping genes or broad-specificity efflux pumps that are not necessarily associated with clinically relevant antibiotic resistance. For example, genes annotated as “glycopeptide resistance” may include general cell envelope biosynthesis genes, and “fluoroquinolone resistance” annotations may reflect DNA gyrase/topoisomerase variants rather than dedicated resistance mechanisms. The actual resistance phenotype must be confirmed through antimicrobial susceptibility testing (e.g., disk diffusion or MIC assays). Fewer than 10 copies were detected for antibiotics such as pleuromutilin, streptogramin, diaminopyrimidine, phosphonic acid, isoniazid, and mupirocin. Sulfonamide, pyrazinamide, and nigericin resistance-associated genes were each represented by only a single copy.

#### 3.5.5. Virulence Factor Profiling and Biosafety Assessment

Annotation against the Virulence Factor Database (VFDB) identified a diverse set of virulence-associated genes across different functional categories in the genome of *B. pumilus* hhy901 (Figure 3D). Genes involved in nutrition and metabolism constituted the largest category (99 copies), followed by immune modulation (85), motility (60), and exotoxin (50). These four categories represented the predominant virulence-related elements. In addition, 29 adhesion-related genes, 28 regulatory system genes, and 28 effector transport system genes were identified. Biofilm formation-associated genes accounted for 20 copies, stress tolerance genes for 12, and extracellular enzyme-encoding genes for 4. Antimicrobial competitive advantage and post-translational modification genes each totaled 3 copies, while 3 genes were assigned to other virulence types. Notably, only a single invasion-associated virulence gene was detected.

It is important to interpret these results with caution. Many genes categorized as “virulence factors” in VFDB correspond to conserved cellular functions that are not necessarily associated with pathogenicity in environmental *Bacillus* species. For example, the 50 genes in the “exotoxin” category may include hemolysin-like proteins, siderophores, and other nutrient-acquisition proteins that are widespread in non-pathogenic bacteria, rather than bona fide toxins. Similarly, “immune modulation” genes may reflect general stress response and metabolic regulators. The predominance of nutrition/metabolism and motility genes is consistent with a free-living, saprophytic lifestyle rather than an invasive pathogenic strategy. The detection of only a single invasion-associated gene suggests a relatively low potential for invasive pathogenicity.

However, the presence of multiple antimicrobial resistance determinants (Section 3.5.4) raises a potential concern regarding horizontal gene transfer (HGT) in environmental settings. Therefore, while genomic analysis suggests a relatively low invasive pathogenic potential, a comprehensive biosafety assessment—including phenotypic antimicrobial susceptibility testing, cytotoxicity assays, and, if warranted, animal model studies—is required before this strain can be considered for environmental application.

## 4. Discussion

This study isolated and characterized a skatole-degrading bacterium, *B. pumilus* hhy901, from bovine manure, and integrated polyphasic taxonomy, degradation assays, and whole-genome sequencing to evaluate its biotechnological potential for livestock manure deodorization. The strain achieved 87.12% removal of 100 mg/L skatole within 96 h, and its genome encodes a broad repertoire of genes potentially associated with aromatic compound degradation, environmental stress resistance, and survival in complex microbial communities. Below, we discuss the significance of these findings in the context of current knowledge, while explicitly distinguishing between experimentally demonstrated phenotypes and genomically inferred potential.

### 4.1. Taxonomic Identification

The polyphasic taxonomic approach—combining 16S rRNA and gyrB gene phylogeny, morphological characterization, biochemical profiling, and whole-cell fatty acid analysis—initially assigned strain hhy901 to *B. pumilus*. However, as noted in the Results, the 16S rRNA gene sequence shared equally high similarity (99.93%) with both *B. pumilus* ATCC 7061T and *B. zhangzhouensis* DW5–4T [21], reflecting the well-documented limitation of 16S rRNA sequencing for species delineation within the *B. pumilus* clade [12]. To resolve the taxonomic position definitively, we employed whole-genome-based approaches including ANI and TYGS dDDH analyses. The ANI value of 95.73% with *B. pumilus* ATCC 7061T exceeded the 95% species delimitation threshold, and the TYGS phylogenomic tree placed hhy901 within the *B. pumilus* clade. Notably, the dDDH d4 value (64.3%) was below the conventional 70% threshold, which likely reflects the substantial accessory genome divergence between hhy901 and the type strain (genome alignment coverage of ~66%). This pattern—ANI above threshold but dDDH below—is occasionally observed in bacterial species with large pan-genomes and high rates of horizontal gene transfer. Nevertheless, the combined weight of evidence from 16S rRNA, gyrB, morphology, biochemistry, fatty acid profiling, ANI, and phylogenomic clustering supports the assignment of hhy901 to *B. pumilus*, while acknowledging its relatively divergent genomic position within the species. This multi-evidence approach ensures taxonomic accuracy and is consistent with current standards for prokaryotic species description [12].

### 4.2. Skatole Degradation Performance and Comparison with Known Bacillus Degraders

The skatole degradation efficiency of strain hhy901 (87.12% removal of 100 mg/L within 96 h) compares favorably with previously reported *Bacillus* strains. A comprehensive review by Xu et al. [4] summarized the current state of microbial skatole degradation, noting that while diverse bacterial genera have been identified, *Bacillus* strains remain relatively underexplored despite their advantageous sporulation and stress resistance properties. Table 4 compares the degradation performance of strain hhy901 with two previously reported *Bacillus* skatole degraders.

As shown in Table 4, strain hhy901 exhibits a substantially higher degradation rate than previously reported *Bacillus* strains, even when accounting for differences in experimental conditions. The higher initial skatole concentration (100 mg/L vs. 10 mg/L) and longer incubation time (96 h vs. 24–48 h) in the present study may partly explain the improved performance, as longer incubation allows for more complete degradation. Nevertheless, the nearly 2-fold higher degradation rate compared with the rumen *Bacillus* isolate (0.908 vs. 0.479 mg/L/h) at the same initial concentration suggests that hhy901 possesses a genuinely stronger skatole-degrading capacity. This may reflect the strain’s adaptation to the high skatole concentrations present in fresh cattle manure, where prolonged environmental selection has favored efficient degraders.

It is important to note that the degradation assays in this study were conducted in nutrient-rich *Bacillus* liquid medium rather than a minimal medium with skatole as the sole carbon source. Therefore, the observed degradation may involve co-metabolic processes, and further experiments in minimal medium are needed to determine whether hhy901 can utilize skatole as a sole carbon and energy source.

### 4.3. Putative Skatole Degradation Pathway

**Figure 4:**
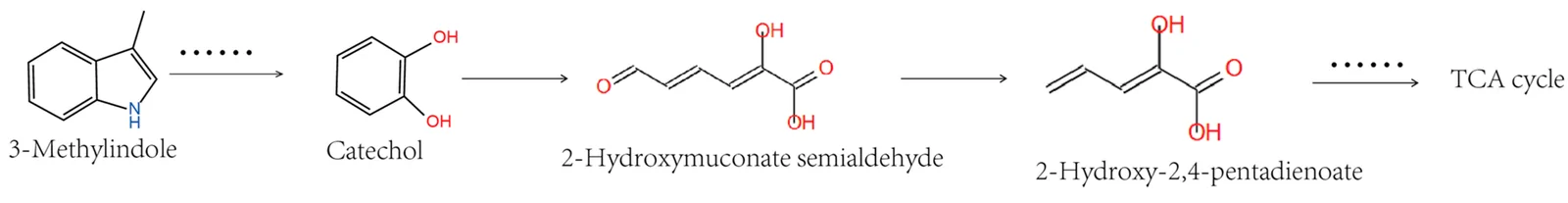
Putative skatole degradation pathway in *B. pumilus* hhy901.

Based on the genomic annotation and known aromatic compound degradation pathways [4,14,22], we propose a putative skatole degradation pathway for *B. pumilus* hhy901 (Figure 4). The pathway is inferred from the presence of relevant genes and has not been experimentally validated; steps supported by genomic evidence are indicated by dashed arrows, while the overall conversion of skatole to biomass and CO_2_ is supported by the degradation assay.

Figure 4. Putative skatole degradation pathway in *B. pumilus* hhy901. Dashed arrows indicate genomically inferred steps that require experimental validation. Key enzymes: CYP, cytochrome P450 monooxygenase; C23O, catechol 2,3-dioxygenase (meta-cleavage); ALDH, aldehyde dehydrogenase; MAO, monoamine oxidase; DmpD, 2-hydroxymuconic semialdehyde hydrolase; PcaC, 4-carboxymuconolactone decarboxylase; PraC, 4-oxalocrotonate tautomerase. TCA, tricarboxylic acid cycle.

In the proposed pathway, skatole (3-methylindole) may first undergo hydroxylation of the pyrrole ring or the benzene ring, catalyzed by cytochrome P450 monooxygenase (cypD_E) or other oxidoreductases, yielding hydroxy-skatole intermediates. The monoamine oxidase gene (aofH) may participate in the oxidative deamination of the indole nitrogen, facilitating ring opening. Subsequent cleavage of the aromatic ring may be catalyzed by catechol 2,3-dioxygenase (catE), which performs meta-cleavage of catechol-like intermediates—a key step in the aerobic degradation of many aromatic pollutants [22,23]. The resulting ring-cleavage products (e.g., 2-hydroxymuconic semialdehyde) may then be processed by enzymes such as DmpD (2-hydroxymuconic semialdehyde hydrolase), PcaC (4-carboxymuconolactone decarboxylase), and PraC (4-oxalocrotonate tautomerase), ultimately channeling carbon into the tricarboxylic acid (TCA) cycle via the phenylacetic acid degradation pathway (paaH, atoB, atoD, atoA) and β-oxidation (fadA, fadB, fadN). Aldehyde dehydrogenase (ALDH) may play a critical role in detoxifying reactive aldehyde intermediates generated during ring cleavage.

This proposed pathway is consistent with the general model of indolic compound degradation described in recent reviews [4] and with the genomic inventory of hhy901. However, several key steps remain speculative. In particular, the initial hydroxylation of skatole and the identity of the first ring-cleavage substrate have not been determined. Future studies should employ transcriptomic analysis (e.g., RNA-seq or RT-qPCR comparing skatole-induced vs. uninduced cells) to identify differentially expressed genes, and metabolomic analysis (e.g., LC-MS/MS or GC-MS) to detect degradation intermediates [14]. Enzyme activity assays for C23O and ALDH in cell-free extracts would further validate the pathway.

### 4.4. Environmental Stress Adaptation

The livestock manure environment presents a complex stress landscape comprising heavy metals, residual veterinary antibiotics, disinfectants, and oxidative stress, all of which can constrain the survival and functionality of inoculated degrading strains [10]. The genome of hhy901 encodes a broad repertoire of genes potentially associated with resistance to these stressors, including 59 nickel resistance-associated genes, 44 tungsten resistance-associated genes, and numerous genes for copper, zinc, cobalt, and arsenic resistance. The abundance of these genes is consistent with the strain’s isolation from manure, where feed-derived trace metals (particularly copper and zinc, commonly used as growth promoters in livestock feed) exert continuous selection pressure [10].

The genome also encodes genes potentially associated with resistance to disinfectants widely used in livestock operations, including quaternary ammonium compounds (QACs), chlorhexidine, and SDS. This is a practically relevant trait, as manure storage facilities and animal housing are routinely disinfected, and a deodorizing inoculant must survive these treatments to function effectively. Similarly, the 21 genes potentially associated with hydrogen peroxide resistance may support survival under oxidative stress conditions in manure.

However, it is critical to emphasize that all of these resistance predictions are based on sequence homology and have not been phenotypically validated. The high copy numbers for certain metals (e.g., 59 nickel genes) likely include multiple paralogous transporter systems with broad substrate specificity rather than dedicated resistance mechanisms. Phenotypic assays—such as minimum inhibitory concentration (MIC) tests for relevant heavy metals and disinfectants—are needed to confirm whether the genomic potential translates into actual tolerance. Until such data are available, claims regarding the strain’s environmental resilience should be regarded as hypotheses rather than conclusions.

### 4.5. Biosafety Assessment

Biosafety is a critical consideration for any microbial strain proposed for environmental release. The genomic analysis of hhy901 identified a relatively low number of invasion-associated virulence genes (only one), and the majority of virulence-factor annotations corresponded to conserved cellular functions such as nutrient acquisition, motility, and biofilm formation—traits that are common in environmental *Bacillus* species and not necessarily indicative of pathogenicity. The 50 genes categorized as “exotoxin” in VFDB likely include hemolysin-like proteins, siderophores, and other nutrient-acquisition factors that are widespread in non-pathogenic bacteria, rather than bona fide toxins. These observations suggest that hhy901 has a relatively low potential for invasive pathogenicity.

That said, several biosafety concerns warrant cautious interpretation. First, the genome encodes a large number of antimicrobial resistance-associated genes across multiple drug classes. While many of these annotations may correspond to conserved housekeeping genes or broad-specificity efflux pumps rather than clinically relevant resistance mechanisms, the presence of even a few transferable resistance genes poses a potential risk of horizontal gene transfer (HGT) to indigenous environmental microbiota or, indirectly, to pathogens. Second, biosafety cannot be determined from genomic data alone; standardized phenotypic assays—including antimicrobial susceptibility testing (e.g., CLSI broth microdilution or disk diffusion), cytotoxicity assays (e.g., against mammalian cell lines), and, if warranted, in vivo virulence testing in appropriate animal models—are required to establish a definitive safety profile.

Therefore, we conclude that while genomic analysis suggests a relatively low invasive pathogenic potential, the strain should be regarded as a candidate for further development rather than a confirmed safe product. We recommend that any future field application be preceded by a comprehensive biosafety assessment following relevant national and international guidelines (e.g., EFSA guidelines for environmental risk assessment of microbial plant protection products, or OECD consensus documents for microbial biosafety).

### 4.6. Practical Implications for Manure Deodorization

The practical application of hhy901 for manure deodorization would require formulation as a stable bio-inoculant. Several properties of *B. pumilus* are advantageous in this regard: (i) the ability to form endospores enables production of shelf-stable spore-based formulations that can withstand high temperatures during storage and transport; (ii) the broad carbohydrate-active enzyme repertoire (108 CAZy genes) may support rapid proliferation on manure polysaccharides, providing the biomass necessary for sustained degradation; (iii) the predicted stress resistance profile may enhance survival in the competitive manure environment.

Potential application strategies include direct inoculation of manure storage pits or lagoons, incorporation into composting systems, or use in biofilters for treating exhaust air from animal housing. For liquid manure systems, a spore-based inoculant could be applied periodically at a defined dosage, while for composting, the strain could be mixed into the compost matrix during the thermophilic phase, leveraging its heat-resistant spores. However, several challenges remain: (i) the strain’s ability to colonize and persist in complex manure microbial communities has not been evaluated; (ii) competition with indigenous microbiota may reduce degradation efficiency in situ; (iii) the optimal dosage, application frequency, and environmental conditions (temperature, pH, moisture) for field performance need to be determined; and (iv) the cost-effectiveness of large-scale production and application must be assessed. Future studies should address these knowledge gaps through pilot-scale trials in real manure management systems.

### 4.7. Study Limitations

This study has several limitations that should be acknowledged:

(1) Lack of functional validation of degradation genes. The proposed skatole degradation pathway is inferred from genomic annotation and has not been experimentally verified. Transcriptomic (e.g., RNA-seq or RT-qPCR), proteomic, or metabolomic (e.g., LC-MS/MS) analyses are needed to confirm which genes are actually expressed and active during skatole degradation, and to identify degradation intermediates.

(2) Degradation assays under nutrient-rich conditions. The skatole degradation experiments were conducted in *Bacillus* liquid medium containing other carbon sources, not in a minimal medium with skatole as the sole carbon source. Therefore, it remains unclear whether hhy901 can utilize skatole as a sole carbon and energy source, or whether degradation occurs via co-metabolism.

(3) No phenotypic validation of stress resistance. All resistance predictions (heavy metals, disinfectants, antibiotics) are based on genomic annotation. Phenotypic susceptibility assays are needed to confirm actual tolerance levels.

(4) Biosafety assessment based solely on genomics. The biosafety evaluation is limited to in silico analysis. Phenotypic safety testing (antimicrobial susceptibility, cytotoxicity, and potentially animal model studies) is required before environmental application.

(5) Laboratory-scale only. All experiments were conducted under controlled laboratory conditions. The strain’s performance in real manure systems, where it must compete with indigenous microbiota and tolerate fluctuating environmental conditions, remains to be evaluated.

(6) No field trials. Pilot-scale or field-scale trials are needed to assess the practical efficacy, persistence, and ecological impact of hhy901 in real livestock production settings.

Future work will focus on (i) transcriptomic and metabolomic validation of the skatole degradation pathway; (ii) phenotypic characterization of stress resistance and biosafety; (iii) optimization of fermentation and formulation processes for spore-based inoculant production; and (iv) pilot-scale field trials in livestock manure management systems.

## 5. Conclusions

In this study, a skatole-degrading bacterium was isolated from bovine manure and identified as *Bacillus pumilus* hhy901 through polyphasic taxonomy, supplemented by genome-based ANI analyses. The strain removed 87.12% of 100 mg/L skatole within 96 h under laboratory conditions, representing a higher degradation rate than previously reported *Bacillus* skatole degraders. Whole-genome sequencing revealed a genome of approximately 3.6 Mb encoding 3564 protein-coding genes, including 39 genes potentially associated with xenobiotic biodegradation, a broad repertoire of stress resistance determinants, and a relatively low number of invasion-associated virulence factors. These genomic features suggest that hhy901 possesses the genetic potential for aromatic compound degradation and environmental adaptation, consistent with its observed skatole-removal phenotype.

However, it is important to emphasize that the functional annotations, degradation pathway model, stress resistance predictions, and biosafety assessment presented in this study are based primarily on genomic inference and require experimental validation. The strain should be regarded as a promising candidate for further development rather than a confirmed bio-deodorization product. Future studies should focus on functional validation of the degradation pathway, phenotypic characterization of stress resistance and biosafety, and pilot-scale field trials to assess practical efficacy. With these validations, *B. pumilus* hhy901 could contribute to the development of sustainable bio-based odor control technologies for intensive livestock production.

## Figures and Tables

**Table 1:** Fatty acid data of strain hhy901.

Peak Name	Percent	Peak Name	Percent
12:0	0.16	15:0 iso 3OH	0.13
13:0 iso	0.30	15:0 2OH	0.12
13:0 anteiso	0.29	17:1 iso ω10c	0.07
14:0 iso	2.21	17:1 iso ω5c	0.06
14:0 anteiso	0.09	17:1 anteiso ω9c	0.15
14:1 ω5c	0.06	17:0 iso	4.91
14:0	0.44	17:0 anteiso	9.23
15:0 iso	29.69	17:0 cyclo	0.17
15:0 anteiso	39.72	16:0 iso 3OH	0.08
16:1 ω7c alcohol	0.06	18:1 ω9c	0.15
16:0 N alcohol	0.06	18:0	0.29
16:0 iso	8.36	17:0 iso 3OH	0.09
16:0	2.59	17:0 2OH	0.10

**Table 2:** API 50CH carbon source utilization profile of strain hhy901.

Analytical Profile Index (API)	Result	Analytical Profile Index (API)	Result
Control	-	Esculin	+
glycerinum	-	Salicoside	+
Erythrose	-	D-Cellobiose	w
D-arabinose	-	D-Maltose	-
L-arabinose	w	D-Lactose	-
D-Ribose	+	D-Melibiose	-
D-xylose	w	D-Sucrose	+
L-xylose	-	D-Trehalose	w
D-Adonitol	-	Inulin	-
β-methyl-D-xyloside	-	D-Melezitose	-
D-Galactose	-	D-Raffinose	-
D-Glucose	+	Starch	-
D-Fructose	+	Glycogen	-
D-Mannose	+	Xylitol	-
L-Sorbose	-	Gentiobiose	w
L-Rhamnose	-	D-Turanose	-
Dulcitol	-	D-Lyxose	-
Inositol	-	D-Tagatose	+
D-Mannitol	+	D-Fucose	-
D-Sorbitol	-	L-Fucose	-
α-methyl-D-mannosidase	-	D-Arabitol	-
α-methyl-D-glucoside	-	L-Arabitol	-
N-Acetylglucosamine	-	Gluconate	-
Amygdalin	w	2-keto-gluconate	-
Arbutin	+	5-keto-gluconate	-

Note: +, positive; -, negative; W, weakly positive.

**Table 3:** Genomic analysis of *Bacillus pumilus* hhy901.

Item	Result
Sample name	*Bacillus pumilus* hhy901
Genome size	3,599,587 bp
Whole genome G+C	41.91%
Gene number	3564
Largest (bp)	25,047
Reads N50(bp)	11,135
Coverage (%) based on K-mer Analysis	101.41

**Table 4:** Comparison of skatole degradation performance between *B. pumilus* hhy901 and previously reported *Bacillus* strains.

Strain	Isolation Source	Initial Skatole (mg/L)	Degradation (%)	Time (h)	Degradation Rate (mg/L/h)	Reference
*Bacillus* sp.	Chicken manure	10	44.5	24	0.185	Xu et al., 2023 [6]
*Bacillus* sp.	Bovine rumen	100	23.03	48	0.479	Wang et al., 2024 [5]
*B. pumilus* hhy901	Bovine manure	100	87.12	96	0.908	This study

Note: Degradation rates were calculated as (initial concentration × degradation percentage)/time. Direct comparison should account for differences in culture medium, temperature, and whether skatole served as the sole carbon source.

## Data Availability

The original contributions presented in this study are included in the article/Supplementary Materials. Further inquiries can be directed to the corresponding authors.

## Supplementary Materials

The following supporting information can be downloaded at: https://www.mdpi.com/article/10.3390/cimb48090964/s1.

## Appendix A

**Rank**	**Name**	**Strain**	**Accession**	**Pairwise Similarity (%)**	**Mismatch/Total nt**
1	*Bacillus pumilus*	ATCC 7061	ABRX01000007	99.93	1/1352
2	*Bacillus zhangzhouensis*	DW5-4	JOTP01000061	99.93	1/1352
3	*Bacillus australimaris*	NH7I_1	JX680098	99.85	2/1352
4	*Bacillus safensis* subsp. *safensis*	FO-36b	ASJD01000027	99.85	2/1352
5	*Bacillus safensis* subsp. *osmophilus*	BC09	KY990920	99.85	2/1352
6	*Bacillus altitudinis*	41KF2b	ASJC01000029	99.56	6/1352
7	*Bacillus xiamenensis*	HYC-10	AMSH01000114	99.48	7/1352
8	*Bacillus atrophaeus*	JCM 9070	AB021181	97.26	37/1352
9	*Bacillus mexicanus*	FSQ1	JAHAWP010000006	97.23	34/1229
10	*Bacillus subtilis*	NCIB 3610	ABQL01000001	97.12	39/1352
11	*Bacillus tequilensis*	KCTC 13622	AYTO01000043	97.04	40/1352
12	*Bacillus siamensis*	KCTC 13613	AJVF01000043	97.04	40/1352
13	*Bacillus stercoris*	JCM 30051	MN536904	97.04	40/1352
14	*Bacillus spizizenii*	NRRL B-23049	CP002905	97.04	40/1352
15	*Bacillus velezensis*	CR-502	AY603658	97.04	40/1351

## References

[1] Qiao M., Nan M., Yujie L., Jingwei W. (2021). Occurrence, impacts, and microbial transformation of 3-methylindole (skatole): A critical review. J. Hazard. Mater..

[2] Barbusinski K., Kalemba K., Kasperczyk D., Urbaniec K., Kozik V. (2017). Biological methods for odor treatment—A review. J. Clean. Prod..

[3] Krzysztof B., Anita P., Damian K. (2021). Removal of Odors (Mainly H2S and NH3) Using Biological Treatment Methods. Clean Technol..

[4] Xu B., Qiu W., Gao X., Ni H., Tao X., Sun L., Lyu W. (2025). Advances in microbial degradation of skatole: A review. Curr. Res. Microb. Sci..

[5] Wang L., Liu M., Zhang F., Ji S., Wang Y., Zhang Y., Duan C., Liu Y., Yan H. (2024). Isolation and Identification of Rumen Skatole-degrading Bacteria and Analysis on Their Degradation Characteristics. Biotechnol. Bull..

[6] Xu X., Zhang H., Liu J., Long H., Qi J., Zhao H., Yu T. (2023). Isolation, screening and effectiveness of deodorizing strains from chicken manure. Heilongjiang Anim. Sci. Vet. Med..

[7] Ding H., Zhao X., Azad M.A.K., Ma C., Gao Q., He J., Kong X. (2021). Dietary supplementation with Bacillus subtilis and xylo-oligosaccharides improves growth performance and intestinal morphology and alters intestinal microbiota and metabolites in weaned piglets. Food Funct..

[8] Monika W., Wojciech Ś., Paweł K., Karol K., Jakub D. (2023). Bioremediation of Heavy Metals by the Genus Bacillus. Int. J. Environ. Res. Public Health.

[9] Jakub D., Zuzanna J., Iryna K., Paweł K., Karol K. (2023). Biocontrol of fungal phytopathogens by Bacillus pumilus. Front. Microbiol..

[10] JinRu F., HongGang N. (2023). Effects of heavy metals and metalloids on the biodegradation of organic contaminants. Environ. Res..

[11] Das S., Dash H.R., Mangwani N., Chakraborty J., Kumari S. (2014). Understanding molecular identification and polyphasic taxonomic approaches for genetic relatedness and phylogenetic relationships of microorganisms. J. Microbiol. Methods.

[12] Michael R., Ramon R.-M., Frank O.G., Jörg P. (2016). JSpeciesWS: A web server for prokaryotic species circumscription based on pairwise genome comparison. Bioinformatics.

[13] Hossain M.S., Iken B., Iyer R. (2024). Whole genome analysis of 26 bacterial strains reveals aromatic and hydrocarbon degrading enzymes from diverse environmental soil samples. Sci. Rep..

[14] Wang Z., Sun J., Yang P., Zhang W., Jiang Y., Liu Q., Yang Y., Hao R., Guo G., Huo W. (2024). Molecular Analysis of Indole and Skatole Decomposition Metabolism in Acinetobacter piscicola p38 Utilizing Biochemical and Omics Approaches. Microorganisms.

[15] Seok-Hwan Y., Sung-Min H., Soonjae K., Jeongmin L., Yeseul K., Hyungseok S., Jongsik C. (2017). Introducing EzBioCloud: A taxonomically united database of 16S rRNA gene sequences and whole-genome assemblies. Int. J. Syst. Evol. Microbiol..

[16] Meier-Kolthoff J.P., Göker M. (2019). TYGS is an automated high-throughput platform for state-of-the-art genome-based taxonomy. Nat. Commun..

[17] Meier-Kolthoff J.P., Carbasse J.S., Peinado-Olarte R.L., Göker M. (2022). TYGS and LPSN: A database tandem for fast and reliable genome-based classification and nomenclature of prokaryotes. Nucleic Acids Res..

[18] Kamimura N., Takahashi K., Mori K., Araki T., Fujita M., Higuchi Y., Masai E. (2017). Bacterial catabolism of lignin-derived aromatics: New findings in a recent decade Update on bacterial lignin catabolism. Environ. Microbiol. Rep..

[19] Valiauga B., Bagdžiūnas G., Sharrock A.V., Ackerley D.F., Čėnas N. (2024). The Catalysis Mechanism of *E. coli* Nitroreductase A, a Candidate for Gene-Directed Prodrug Therapy: Potentiometric and Substrate Specificity Studies. Int. J. Mol. Sci..

[20] Singh R., Shahul R., Kumar V., Yadav A.K., Mehta P.K. (2025). Microbial amidases: Characterization, advances and biotechnological applications. Biotechnol. Notes.

[21] Liu Y., Lai Q., Du J., Shao Z. (2016). *Bacillus zhangzhouensis* sp. nov. and *Bacillus australimaris* sp. nov. Int. J. Syst. Evol. Microbiol..

[22] Lubbers R.J.M. (2025). An Updated Perspective on the Aromatic Metabolic Pathways of Plant-Derived Homocyclic Aromatic Compounds in Aspergillus niger. Microorganisms.

[23] Yamilet M.B.G., Daniel R.T.J., Alberto H.E.J., Carlos O.P., Jeiry T.J., Ángel R.B.M., Erubiel T.H., Augusto R.A., Yanet R.R. (2023). The catE gene of Bacillus licheniformis M2-7 is essential for growth in benzopyrene, and its expression is regulated by the Csr system. World J. Microbiol. Biotechnol..

