# Supplementary material for: Genomic Characterization and Biotechnological Potential of a Skatole-Degrading Bacillus pumilus Strain hhy901 Isolated from Bovine Manure
Source: Curr Issues Mol Biol. 2026 Sep 20;48(9):964. doi: 10.3390/cimb48090964 (PMC13605943; doi:10.3390/cimb48090964)
Supplement: Supplementary file 1 [file cimb-48-00964-s001.zip › Table S1.pdf]

Table S1：Time course of skatole degradation by *Bacillus pumilus* hhy901.

	**0 h**	**12 h**	**24 h**	**48 h**	**72 h**	**96 h**
C_0 (mg/L)	100					
C_{ck} (mg/L)	100	99.86	98.95	97.55	94.8	90.71
C_{ck} (mg/L)	100	99.85	99.01	96.98	94.43	91.01
C_{ck} (mg/L)	100	99.89	99.14	96.7	94.51	91.1
C_{ck} (mg/L) average	100	99.87	99.03	97.08	94.58	90.94
C_{t-1} (mg/L)	100	87.75	78.38	56.79	33.63	4.98
C_{t-2} (mg/L)	100	88.77	77.05	54.39	31.52	2.96
C_{t-3} (mg/L)	100	88.19	78.66	55.64	29.12	3.52
C_0-C_{t-1} (mg/L)	0	12.12	20.65	40.29	60.95	85.96
C_0-C_{t-2} (mg/L)	0	11.1	21.98	42.69	63.06	87.98
C_0-C_{t-3} (mg/L)	0	11.68	20.37	41.44	65.46	87.42
$[(C_0-C_{t-1}) / C_0] \times 100$ % (%)	0	12.12	20.65	40.29	60.95	85.96
$[(C_0-C_{t-2}) / C_0] \times 100$ % (%)	0	11.1	21.98	42.69	63.06	87.98
$[(C_0-C_{t-3}) / C_0] \times 100$ % (%)	0	11.68	20.37	41.44	65.46	87.42
average (%)	0	11.63	21.00	41.47	63.16	87.12
Degradation Mean ± SD (%)	0.00 ± 0.00	11.63 ± 0.51	21.00 ± 0.86	41.47 ± 1.20	63.16 ± 2.26	87.12 ± 1.04

Note: C_0 is the initial skatole concentration (100 mg/L). C_{ck} is the skatole concentration in the abiotic blank control (no bacterial inoculum) measured at 0, 12, 24, 48, 72, and 96 h. C_t is the skatole concentration in the treatment group (inoculated with hhy901), corrected for abiotic loss by subtracting (100 − C_{ck} average) from the raw measured value.
